# Facile Synthesis of Zn/Fe@C Photocatalyst for Visible-Light-Driven H_2_O_2_ Production and Pollutant Degradation

**DOI:** 10.3390/nano16191255

**Published:** 2026-10-04

**Authors:** Osama K. Basalam, Majed S. Almalki, Muhammad Nadeem Arshad, Sher Bahadar Khan, Kalsoom Akhtar, Nujud Maslamani, Esraa M. Bakhsh

**Affiliations:** 1 Chemistry Department, Faculty of Science, King Abdulaziz University, P.O. Box 80203, Jeddah 21589, Saudi Arabia; 2 Center of Excellence for Advanced Materials Research, King Abdulaziz University, P.O. Box 80203, Jeddah 21589, Saudi Arabia; 3 Nanotechnology Research Unit, College of Science, Jazan University, P.O. Box. 114, Jazan 45142, Saudi Arabia; 4 Department of Physical Sciences, Chemistry Division, College of Science, Jazan University, P.O. Box. 114, Jazan 45142, Saudi Arabia

**Keywords:** Zn/Fe@C, photocatalysis, hydrogen peroxide production, dye degradation

## Abstract

A zinc oxide–iron oxide embedded graphitic carbon (Zn/Fe@C) photocatalyst was successfully synthesized by pyrolysis of chitosan hydrogel impregnated with zinc and iron salts. Structural characterization confirmed the formation of a predominantly crystalline, multi-phase oxide system, while morphological analysis revealed a rough and heterogeneous surface with aggregated features. Elemental analysis further verified the incorporation of Zn and Fe species within the graphitic carbon matrix. The photocatalytic performance of the prepared material was evaluated through hydrogen peroxide (H_2_O_2_) production and dye degradation under simulated solar irradiation. Zn/Fe@C exhibited superior H_2_O_2_ production performance, achieving a maximum concentration of 2.9 mmol L^−1^. The use of different alcohols as sacrificial agents revealed that 2-propanol significantly enhanced H_2_O_2_ generation, while increasing catalyst loading improved H_2_O_2_ production due to the availability of additional active sites. In dye degradation experiments, the photocatalyst demonstrated high efficiency toward the removal of representative organic dyes, including methylene blue, methyl orange, and acridine orange, with the latter showing the highest degradation efficiency of 96.6%. The effect of catalyst loading indicated that lower dosages resulted in optimal performance, which was attributed to improved light penetration and reduced aggregation effects. Control experiments confirmed that photocatalysis was the dominant mechanism over adsorption. Furthermore, increasing the metal precursor concentration enhanced photocatalytic activity, while the material maintained good reusability over multiple cycles. Furthermore, Zn/Fe@C was compared with Cu/Fe@C and Cu/Zne@C where Zn/Fe@C exhibited superior performance among the investigated photocatalysts in H_2_O_2_ production and dye degradation. Overall, the results highlight the potential of the developed Zn/Fe@C photocatalyst as a promising material for sustainable H_2_O_2_ production and efficient degradation of organic pollutants.

## 1. Introduction

The rapid growth of industrial and urban activities has led to the continuous discharge of hazardous pollutants into aquatic environments, posing significant threats to ecosystems and human health. Among these contaminants, synthetic dyes are widely recognized as one of the most persistent and problematic classes due to their complex aromatic structures, high chemical stability, and resistance to conventional treatment processes [1,2,3]. The presence of dyes in wastewater not only affects water quality and light penetration but also introduces toxic and potentially carcinogenic compounds into the environment. As a result, the development of efficient and sustainable methods for dye removal has become a critical research priority.

Photocatalysis has emerged as an advanced oxidation process capable of addressing these challenges through the utilization of light energy to generate reactive species for pollutant degradation [3,4]. Metal oxide-based photocatalysts, such as TiO_2_, ZnO, and Fe-based materials, have been extensively studied for their ability to degrade a wide range of organic pollutants under irradiation [2,5]. Despite their advantages, these materials often suffer from limitations including rapid electron–hole recombination and restricted activity under visible light, which significantly reduce their overall efficiency. Consequently, recent efforts have focused on modifying photocatalysts to improve light absorption and enhance charge separation.

In addition to pollutant degradation, hydrogen peroxide (H_2_O_2_) has attracted increasing attention as an environmentally friendly oxidant with applications in water treatment, disinfection, and chemical synthesis [6,7]. Compared to conventional industrial production methods, photocatalytic generation of H_2_O_2_ offers a more sustainable and energy-efficient alternative, as it can proceed under mild conditions using oxygen and light as primary inputs [8,9,10,11]. Various photocatalytic systems, including graphitic carbon nitride, covalent organic frameworks and metal oxide composites, have demonstrated promising performance for solar-driven H_2_O_2_ production [12,13,14,15]. Moreover, iron-based materials have been shown to play a key role in facilitating oxygen reduction pathways leading to H_2_O_2_ formation [16].

To further improve photocatalytic performance, the use of carbon-based materials has gained considerable interest due to their excellent electrical conductivity, high surface area, and ability to enhance charge transport [17,18,19]. In particular, nitrogen-doped carbon materials derived from chitosan have demonstrated significant potential in oxygen reduction reactions and H_2_O_2_ generation systems [17,18,19]. These materials provide abundant active sites and promote efficient electron transfer, thereby improving photocatalytic activity.

Chitosan, a naturally derived biopolymer, has emerged as a versatile and sustainable support material for photocatalyst design due to its biodegradability, non-toxicity, and functional groups capable of coordinating with metal ions [20,21]. Chitosan-based composites have been successfully applied in photocatalytic systems for dye degradation, particularly when combined with metal oxides such as ZnO and Fe_2_O_3_ [22,23]. The incorporation of metal species into chitosan matrices enhances adsorption capacity and provides improved dispersion of active sites, which contributes to higher catalytic efficiency. Additionally, chitosan-derived materials have been utilized to produce hybrid nanocomposites with enhanced photocatalytic and antibacterial properties [24,25].

Furthermore, several studies have demonstrated the effectiveness of chitosan-based composites in advanced oxidation processes such as photo-Fenton systems for dye degradation [26]. The combination of multiple metal oxides within a single system has also been explored as a strategy to improve photocatalytic activity through synergistic interactions [21,22]. These multi-component systems can facilitate charge separation and enhance the generation of reactive oxygen species (ROS), which are essential for efficient pollutant degradation.

Despite these advancements, the development of multifunctional photocatalysts capable of simultaneously addressing pollutant degradation and H_2_O_2_ production remains limited. In particular, the integration of Zn and Fe species within chitosan-derived carbon matrices for dual photocatalytic applications has not been extensively investigated. Moreover, the influence of synthesis parameters, such as metal precursor concentration, on the structural and photocatalytic properties of such systems requires further exploration.

In this study, Zn/Fe@C was synthesized via a straightforward approach involving hydrogel formation, metal ion impregnation, and thermal treatment under an inert atmosphere. The prepared material was systematically evaluated for its photocatalytic performance in hydrogen peroxide production and the degradation of representative organic dyes under simulated solar irradiation. The effects of operational parameters, including catalyst loading, type of sacrificial agent, and metal precursor concentration, were investigated in detail. Additionally, the reusability and stability of the photocatalyst were assessed, and a possible photocatalytic mechanism was proposed based on the experimental findings.

## 2. Materials and Methods

### 2.1. Materials

All chemicals and reagents used in this study were of analytical grade and were used as received from the respective suppliers without further purification. Chitosan was obtained from Pure-lab Chem Industries, and acetic acid was purchased from Atom Scientific, while sodium hydroxide (NaOH) was purchased from POCH SA. Zinc sulfate heptahydrate (ZnSO_4_·7H_2_O) and iron(II) chloride (FeCl_3_.6H_2_O), used as precursors for the preparation of Zn/Fe@C, were supplied by BDH Laboratory Reagents. Copper(II) chloride (CuCl_2_) and zinc chloride (ZnCl_2_), used for the preparation of Cu/Zn@C, were obtained from Sigma-Aldrich, whereas copper(II) nitrate [Cu(NO_3_)_2_] and iron(II) sulfate (FeSO_4_), used for the preparation of Cu/Fe@C, were supplied by BDH Laboratory Reagents. Methanol (MeOH), ethanol (EtOH), and 2-propanol (2-PrOH), used as sacrificial agents during the photocatalytic H_2_O_2_ production experiments, were obtained from Fisher Scientific. Potassium iodide (KI) was purchased from Sigma-Aldrich, while potassium hydrogen phthalate (KHP), used for the colorimetric determination of H_2_O_2_, was obtained from Loba Chemie Pvt. Ltd. Methylene blue (MB) was supplied by Loba Chemie Pvt. Ltd., whereas methyl orange (MO) and acridine orange (AO), used as model organic dyes in the photocatalytic degradation experiments, were obtained from Sigma-Aldrich. Laboratory-produced deionized water was used throughout all synthesis and photocatalytic experiments.

### 2.2. Synthesis of Zn/Fe@C Photocatalyst

**Scheme 1:**
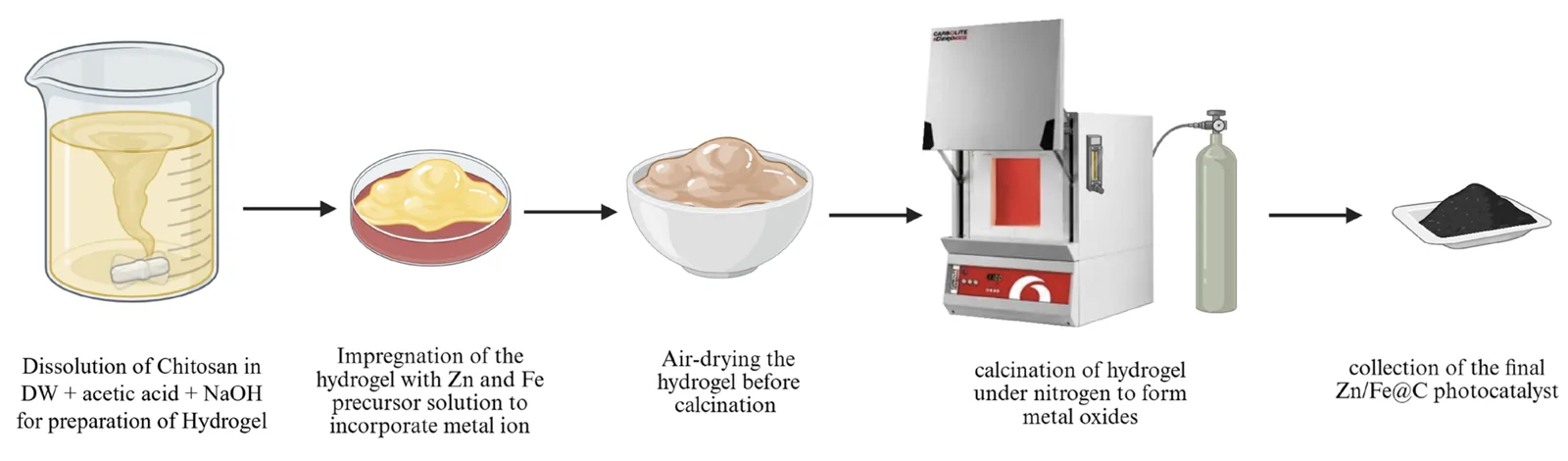
Schematic representation for the synthesis route of the Zn/Fe@C photocatalyst via chitosan dissolution, hydrogel formation, metal ion impregnation, and calcination under nitrogen atmosphere.

The Zn/Fe@C photocatalyst was synthesized using chitosan as a biopolymeric support for Zn/Fe metal precursors (Scheme 1). Chitosan was first dispersed in deionized water, followed by the addition of approximately 10 mL of acetic acid to facilitate its dissolution. The mixture was stirred at room temperature for 4 h until a homogeneous viscous chitosan solution with a concentration of 10% (*w*/*v*) was obtained. The resulting chitosan solution was then added to a 4 mol L^−1^ NaOH solution to induce gelation and form the chitosan hydrogel through alkaline neutralization [27]. For metal loading, separate 3 mol L^−1^ and 5 mol L^−1^ stock solutions of ZnSO_4_ and FeCl_3_ were first prepared and then mixed together at a 1:1 molar ratio, i.e., 3 mol L^−1^ ZnSO_4_ + 3 mol L^−1^ FeCl_3_ and 5 mol L^−1^ ZnSO_4_ + 5 mol L^−1^ FeCl_3_ mixed solutions. A total volume of 100 mL of the mixed metal precursor solution was used for each preparation. The entire chitosan hydrogel was then fully immersed in the mixed Zn/Fe solutions and kept under static conditions in a covered vessel at room temperature for overnight to allow sufficient diffusion and incorporation of the metal species into the chitosan matrix. A visible color change was observed after the immersion step, indicating successful interaction between the hydrogel and the metal precursor solution. The chitosan hydrogel immersed in 100 mL of 3 mol L^−1^ ZnSO_4_ + 3 mol L^−1^ FeCl_3_ and 5 mol L^−1^ ZnSO_4_ + 5 mol L^−1^ FeCl_3_ mixed solutions was denoted as Zn/Fe@C-3M and Zn/Fe@C-5M, respectively. After impregnation, the metal-loaded hydrogel was collected, washed with deionized water 2–3 times to remove loosely bound species, and dried in an oven at 80 °C until a fully solid material was obtained. The dried precursor was then calcined at 900 °C for 5 h under a continuous nitrogen atmosphere to convert the metal-loaded chitosan matrix into the corresponding metal oxide embedded carbon photocatalyst. The final calcined product was obtained as a powder. The same synthesis procedure was applied for both precursor concentrations, with the only variation being the concentration of the Zn/Fe precursor solution. The overall preparation method was carried out in aqueous medium, at ambient-temperature processing conditions during the synthesis stage, and without the use of toxic organic solvents, highlighting the environmentally friendly nature of the adopted route. For comparison, Cu/Zn@C and Cu/Fe@C were prepared using the same synthesis procedure and a precursor concentration of 3 mol L^−1^ for each metal salt. CuCl_2_ and ZnCl_2_ were used as the metal precursors for Cu/Zn@C, whereas Cu(NO_3_)_2_ and FeSO_4_ were used for Cu/Fe@C. In both cases, the two metal precursor solutions were combined at a 1:1 molar ratio. All subsequent impregnation, washing, drying, and thermal treatment conditions were maintained consistently with those used for Zn/Fe@C-3M. In addition, a higher precursor concentration was investigated only for the Zn/Fe@C system to examine the influence of metal precursor concentration on AO removal performance. For this purpose, Zn/Fe@C-5M was prepared using 5 mol L^−1^ ZnSO_4_ + 5 mol L^−1^ FeCl_3_ under identical synthesis conditions.

### 2.3. Analytical Methods

X-ray diffraction (XRD) analysis was used to examine the crystalline structure and identify the crystalline phases of the selected Zn/Fe@C photocatalyst. The diffraction pattern was recorded using Cu Kα radiation with a wavelength (λ) of 1.54060 Å. The observed diffraction peaks were compared with standard Powder Diffraction File (PDF) reference patterns for phase identification. The crystallite size was estimated using the Scherrer equation, D = Kλ/(βcosθ), where D is the crystallite size, K is the shape factor (0.9), λ is the X-ray wavelength, β is the full width at half maximum (FWHM) expressed in radians, and θ is the Bragg angle. The surface morphology of the selected Zn/Fe@C photocatalyst was examined using scanning electron microscopy (SEM). The SEM images were used to observe the surface features, particle morphology, and agglomeration of the prepared material. Energy-dispersive X-ray spectroscopy (EDX), coupled with SEM, was used to determine the elemental composition and confirm the presence of Zn and Fe in the prepared photocatalyst. Attenuated Total Reflectance–Fourier-Transform Infrared (ATR-FTIR) spectroscopy was used to examine the functional groups and chemical bonding characteristics of the selected Zn/Fe@C photocatalyst. The obtained spectrum was analyzed based on the positions of the characteristic absorption bands to identify the main surface functional groups and metal–oxygen-related vibrations present in the prepared material. The optical absorption behavior of the solid Zn/Fe@C photocatalyst was evaluated using UV–Vis spectroscopy. The recorded UV–Vis absorption data were subsequently used to estimate the optical band-gap energy using the Tauc approach [28]. The photon energy (hv) was calculated from the corresponding wavelength according to: hv = 1240/λ where hv is the photon energy expressed in eV and λ is the wavelength in nm. Assuming a direct allowed optical transition, the band-gap energy was evaluated using the Tauc relation:

(αhv)^2^ = A(hv − E_g_)

where α is the absorption coefficient, A is a proportionality constant, and E_g_ is the optical band-gap energy. Accordingly, (αhv)^2^ was plotted as a function of hv, and the linear portion near the absorption edge was extrapolated to (αhv)^2^ = 0. The intercept with the photon-energy axis was taken as the estimated optical band-gap energy of Zn/Fe@C.

### 2.4. Photocatalytic Experiments

The photocatalytic performance of the prepared Zn/Fe@C photocatalysts was evaluated through hydrogen peroxide (H_2_O_2_) production and dye photodegradation experiments under simulated solar irradiation using a xenon short-arc lamp (1000 W) positioned at a distance of 10 cm from the reaction system. All experiments were conducted under ambient conditions and open-air atmosphere.

#### 2.4.1. Hydrogen Peroxide Production

**Scheme 2:**
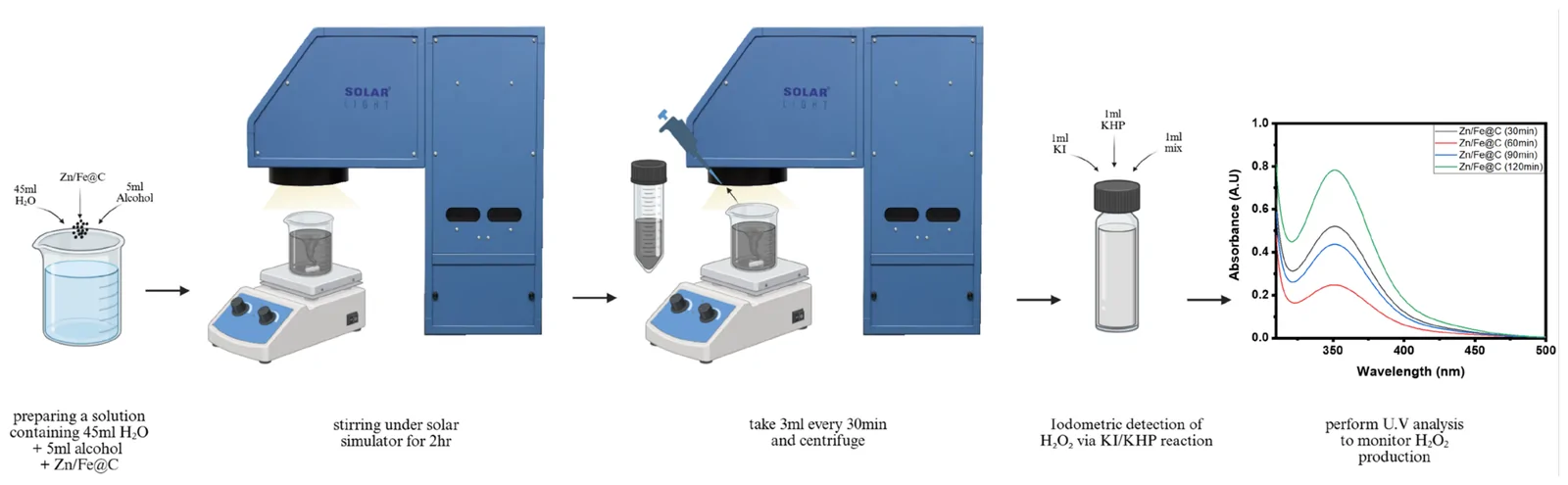
Schematic representation of the experimental setup and procedure for H_2_O_2_ production over Zn/Fe@C under visible light irradiation.

Photocatalytic H_2_O_2_ production experiments were carried out in a beaker-type reactor containing a total reaction volume of 50 mL, composed of 45 mL deionized water and 5 mL alcohol. Methanol, ethanol, and 2-propanol were individually employed as sacrificial agents to investigate the effect of alcohol type on photocatalytic H_2_O_2_ production. The photocatalyst was added at different dosages (5, 10, and 20 mg), and the suspension was exposed to continuous irradiation under stirring (Scheme 2).

The reaction was performed for 120 min, and aliquots were collected at regular intervals (30, 60, 90, and 120 min). After irradiation, the collected samples were centrifuged to remove the photocatalyst particles. For H_2_O_2_ quantification, a colorimetric method was employed by mixing 1 mL of the treated solution with 1 mL of potassium iodide (KI, 0.4 mol L^−1^) and 1 mL of potassium hydrogen phthalate (KHP, 0.1 mol L^−1^). The mixture was allowed to react for 30 min, after which the absorbance was measured using UV–Vis spectroscopy. The concentration of H_2_O_2_ was determined using calibration curve based on the Beer–Lambert law.

#### 2.4.2. Dye Photodegradation

**Scheme 3:**
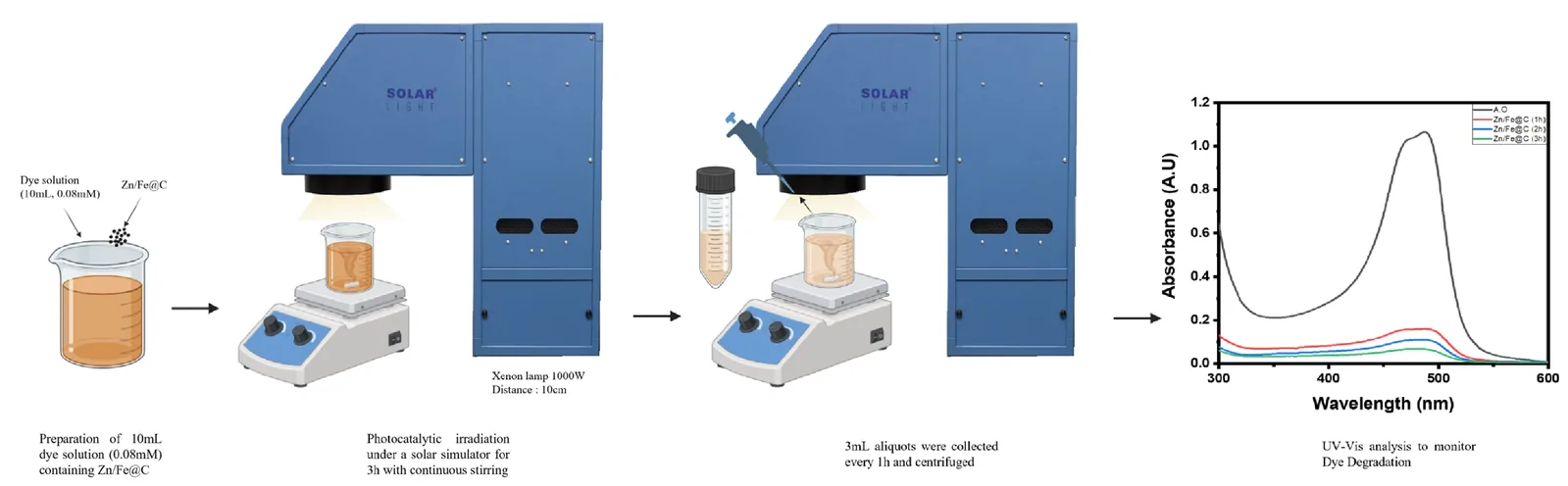
Schematic representation of the experimental setup and procedure for photocatalytic dye degradation using Zn/Fe@C.

The photocatalytic degradation of representative organic dyes, namely MB, MO, and AO, was investigated under similar irradiation conditions (Scheme 3). Each experiment was conducted using 10 mL of dye solution with an initial concentration of 0.08 mmol L^−1^. The initial concentration was selected as a screening condition to evaluate the photocatalytic performance of Zn/Fe@C and to provide consistent conditions for comparing the degradation behavior of the three model dyes [29]. The photocatalyst was added at dosages of 5, 10, and 20 mg. The reaction mixtures were irradiated for 3 h under continuous stirring without a prior dark adsorption step. Samples were collected at regular time intervals (every 1 h), followed by centrifugation to remove the catalyst before analysis. A separate dark-control experiment was conducted using AO and Zn/Fe@C under comparable conditions to independently evaluate the contribution of adsorption to the overall dye removal. This dark-control experiment was not used as a pre-equilibration step for the photocatalytic experiments. The residual dye concentration was determined using UV–Vis spectroscopy at their respective maximum absorption wavelengths (λ_max_): ≈668 nm for MB, ≈460–470 nm for MO, and ≈490 nm for AO. The degradation efficiency was calculated based on the relative decrease in absorbance using the standard percentage removal equation.

The reusability and operational stability of the prepared Zn/Fe@C photocatalyst were evaluated based on the photocatalytic degradation of AO. The experiments were conducted over three consecutive cycles under identical reaction conditions, including catalyst dosage, initial dye concentration, light source, and irradiation time. After each cycle, the photocatalyst was recovered by filtration, thoroughly washed with deionized water to remove residual reactants and intermediates, and then air-dried before being reused in the subsequent run. The photocatalytic performance in each cycle was assessed in terms of degradation efficiency (%).

## 3. Results and Discussion

### 3.1. Characteristics Properties of Zn/Fe@C

#### 3.1.1. Structural and Phase Analysis (XRD)

**Figure 1:**
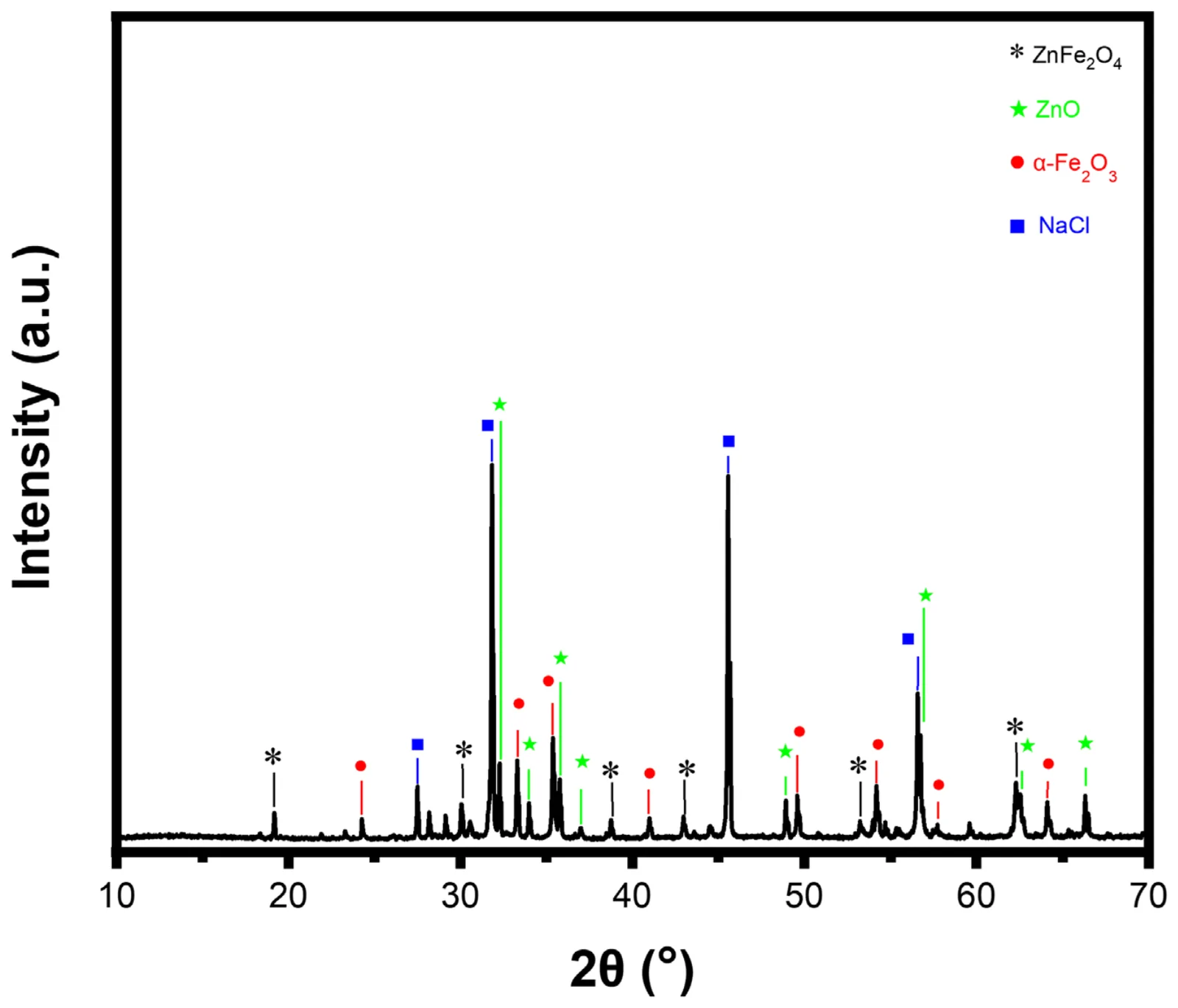
XRD pattern of the Zn/Fe@C photocatalyst.

The X-ray diffraction (XRD) pattern of the calcined Zn/Fe@C photocatalyst is presented in Figure 1. The diffraction profile exhibits several well-defined and sharp peaks, indicating the predominantly crystalline nature of the prepared material. The observed crystallinity can be attributed to the thermal treatment at 900 °C. The XRD pattern of the calcined Zn/Fe@C material is shown in Figure 1. The diffraction pattern reveals a crystalline and multiphase structure. Several reflections observed at approximately 24.23°, 33.24°, 40.92°, 49.53°, and 54.14° can be assigned to α-Fe_2_O_3_ (hematite), in good agreement with the characteristic diffraction peaks reported for the hematite phase [30]. Additional reflections observed at approximately 19.12°, 30.01°, 38.70°, 42.90°, 53.18°, and 62.36° are consistent with reflections associated with the spinel ZnFe_2_O_4_ phase. Several additional reflections, particularly those observed around 31.77°, 35.71–36.92°, 48.14°, and 56.51°, occur close to characteristic reflections reported for hexagonal wurtzite ZnO [31]. These reflections suggest the possible formation of a ZnO-containing phase during calcination. The coexistence of ZnFe_2_O_4_ with α-Fe_2_O_3_ and ZnO has also been reported in thermally treated Zn–Fe oxide systems [32], supporting the possible formation of a zinc ferrite phase during calcination. In addition, reflections observed at approximately 27.43°, 31.77°, 45.50°, and 56.51° are consistent with the characteristic (111), (200), (220), and (222) reflections of crystalline NaCl, respectively [33]. The presence of NaCl may be associated with residual sodium- and chloride-containing species introduced during the synthesis, considering the use of NaOH during chitosan hydrogel formation and FeCl_3_ as the iron precursor. Although Na was not detected by EDX, a minor or heterogeneously distributed crystalline NaCl phase may not necessarily be represented in the localized region examined by EDX. The absence of a dominant broad diffraction feature associated with the chitosan precursor suggests substantial transformation of the polymeric matrix during calcination. The apparent crystallite size was estimated from the representative reflection at approximately 33.25° using the Scherrer equation, yielding a value of approximately 83 nm. Since instrumental broadening was not independently determined, this value should be regarded as an approximate crystallite-size estimate. Overall, the XRD results indicate the formation of a crystalline, multiphase Zn/Fe-containing material following thermal treatment of the chitosan-based precursor.

#### 3.1.2. Morphological Analysis (SEM)

**Figure 2:**
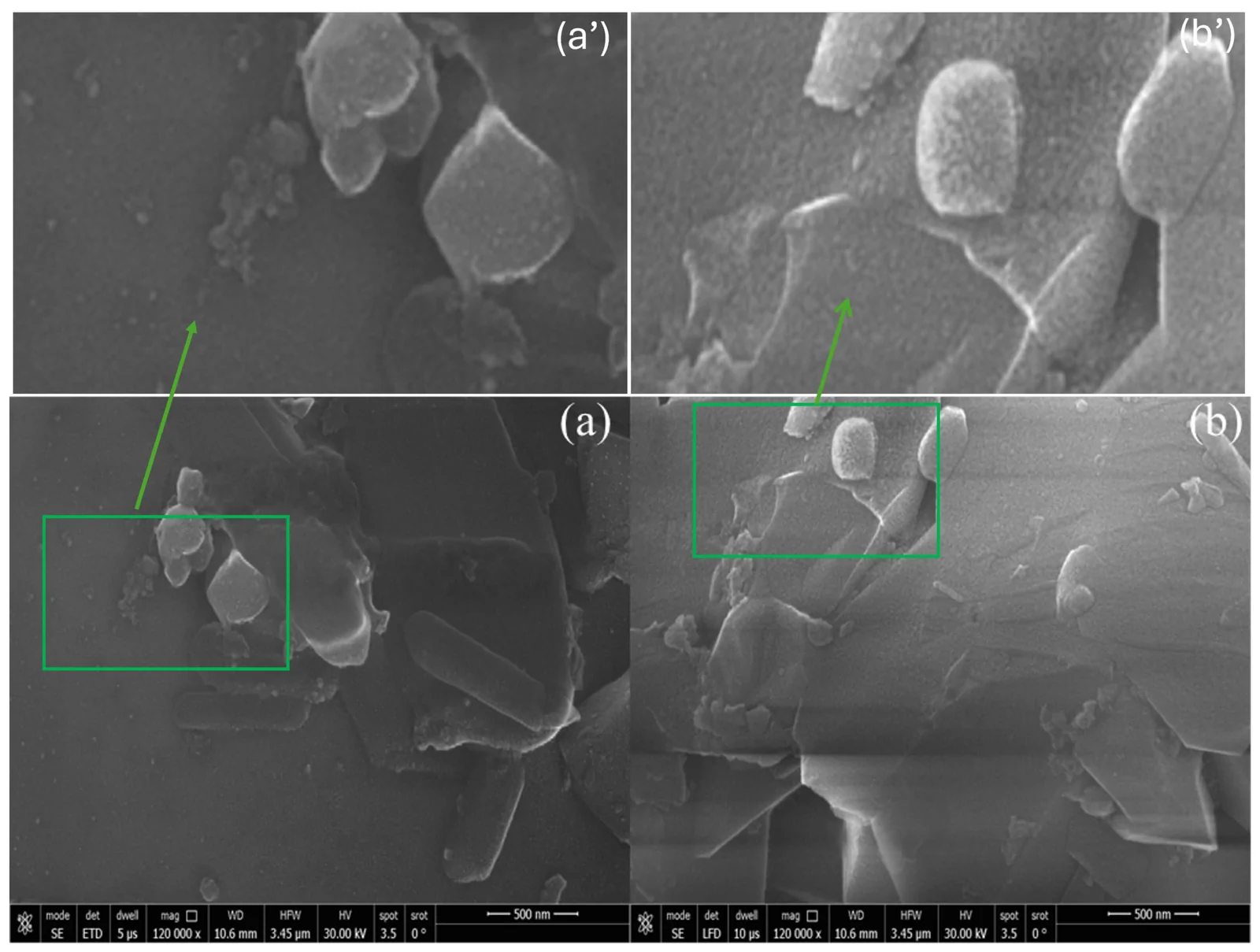
(**a**,**b**) SEM images of the Zn/Fe@C photocatalyst, (**a′**) magnetified picture of the encircle area of image (**a**) and (**b′**) magnetified picture of image (**b**).

The surface morphology of the Zn/Fe@C photocatalyst was examined using scanning electron microscopy (SEM), as shown in Figure 2a,b. The images reveal the formation of irregular, plate-like structures with submicron dimensions, indicating the development of non-uniform particle morphology after calcination. A noticeable degree of particle aggregation is observed in (Figure 2a′,b′) (zoomed encircle area of Figure 2a,b), which may be associated with the thermal treatment applied during calcination. Despite this aggregation, the material exhibits a relatively rough and heterogeneous surface. In addition, smaller features are distributed over larger structures, suggesting a non-uniform distribution of the inorganic components within the chitosan-derived carbon matrix. Overall, the SEM analysis suggests the formation of an aggregated, rough, and heterogeneous surface structure, which is considered beneficial for photocatalytic applications.

#### 3.1.3. Elemental Analysis (EDX)

**Figure 3:**
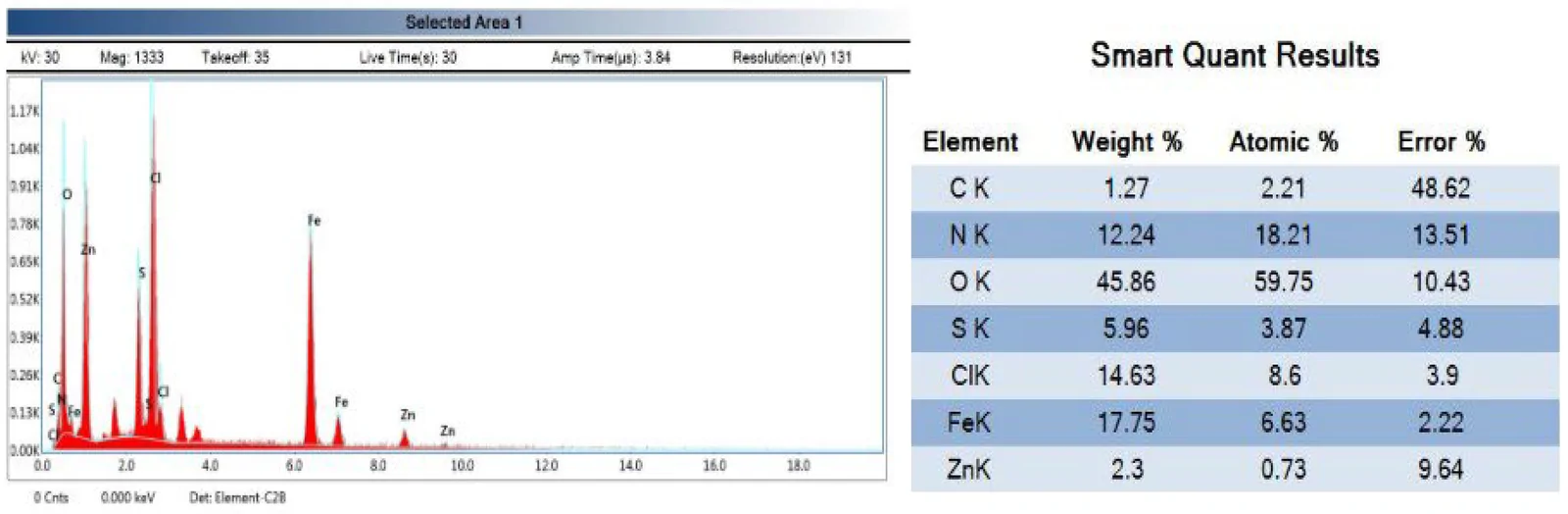
EDX spectrum of the Zn/Fe@C photocatalyst.

The elemental composition of the prepared Zn/Fe@C photocatalyst was analyzed using energy-dispersive X-ray spectroscopy (EDX), as shown in Figure 3. The spectrum confirms the presence of the main constituent elements, including carbon (C), oxygen (O), iron (Fe), and zinc (Zn), indicating the formation of oxide-based structures.

In addition, the detection of nitrogen (N) can be attributed to the chitosan-derived matrix, suggesting the partial retention or transformation of nitrogen-containing functional groups after calcination. Minor signals corresponding to chlorine (Cl) and sulfur (S) are also observed, which may originate from the metal precursors used during synthesis.

The presence of these elements without any unexpected impurities supports the successful incorporation of Zn and Fe species within the chitosan-derived carbon framework. Overall, the EDX results are consistent with the XRD findings and confirm the formation of the Zn/Fe@C photocatalyst.

#### 3.1.4. ATR-FTIR Analysis

**Figure 4:**
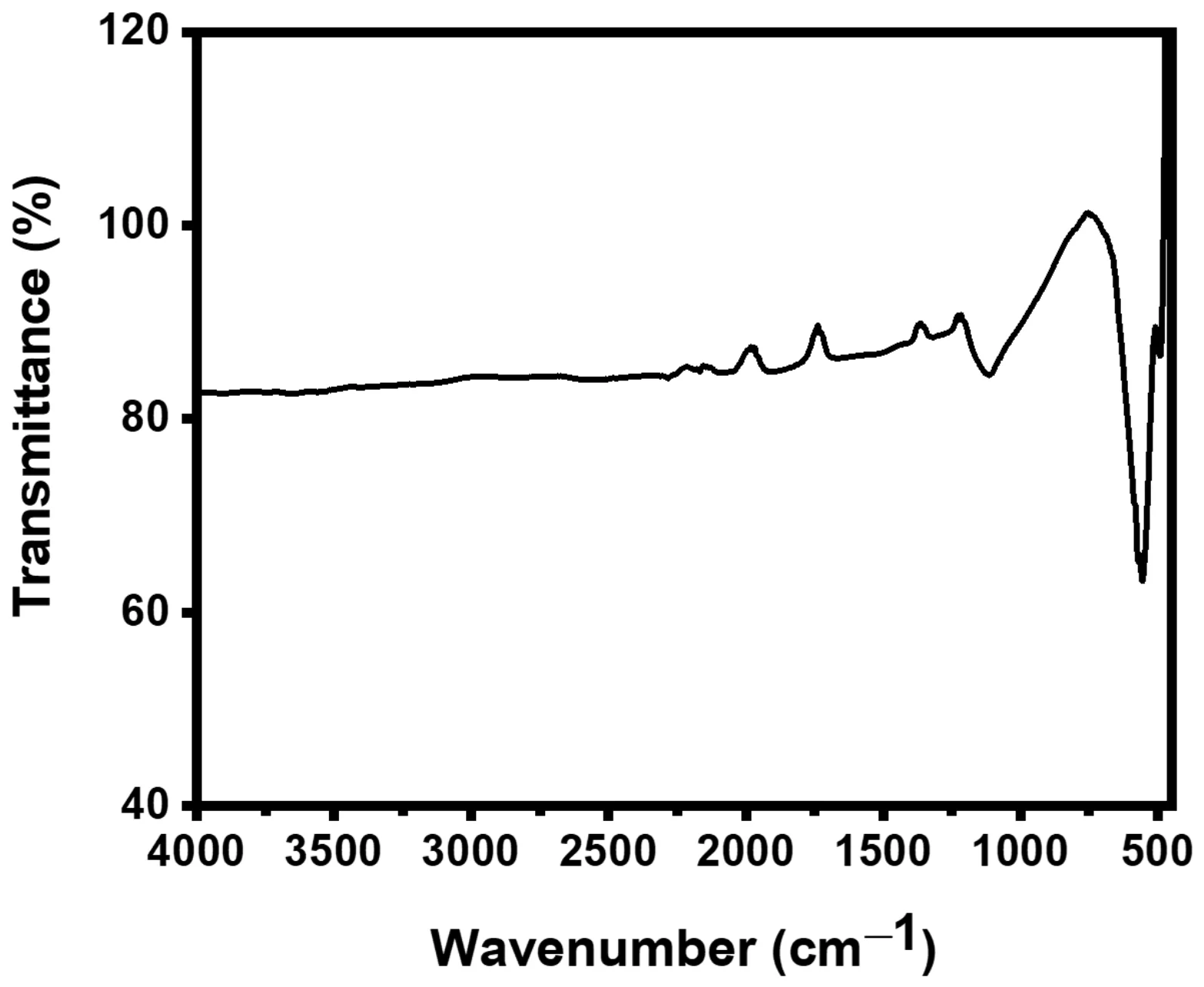
ATR-FTIR spectrum of the calcined Zn/Fe@C photocatalyst.

The ATR-FTIR spectrum of the calcined Zn/Fe@C photocatalyst is presented in Figure 4. The spectrum exhibits a pronounced absorption feature in the low-wavenumber region, particularly below approximately 700 cm^−1^. Absorption bands in this region are commonly associated with metal–oxygen lattice vibrations in Zn–Fe-containing oxides and ferrite materials, including Zn–O and Fe–O vibrations [34,35]. These spectral features are therefore consistent with the presence of Zn- and Fe-containing inorganic components and complement the phase and elemental information obtained from the XRD and EDX analyses. An additional absorption feature is observed in the region of approximately 1000–1300 cm^−1^, which may be associated with residual oxygen-containing surface species and/or other components of the multiphase material. In contrast, the higher-wavenumber region exhibits relatively weak spectral features, suggesting substantial alteration of the original chitosan functional groups following thermal treatment at 900 °C. Overall, the ATR-FTIR results provide complementary evidence for the formation of a calcined Zn/Fe-containing inorganic/carbon-derived material.

#### 3.1.5. Band Gap Analysis

**Figure 5:**
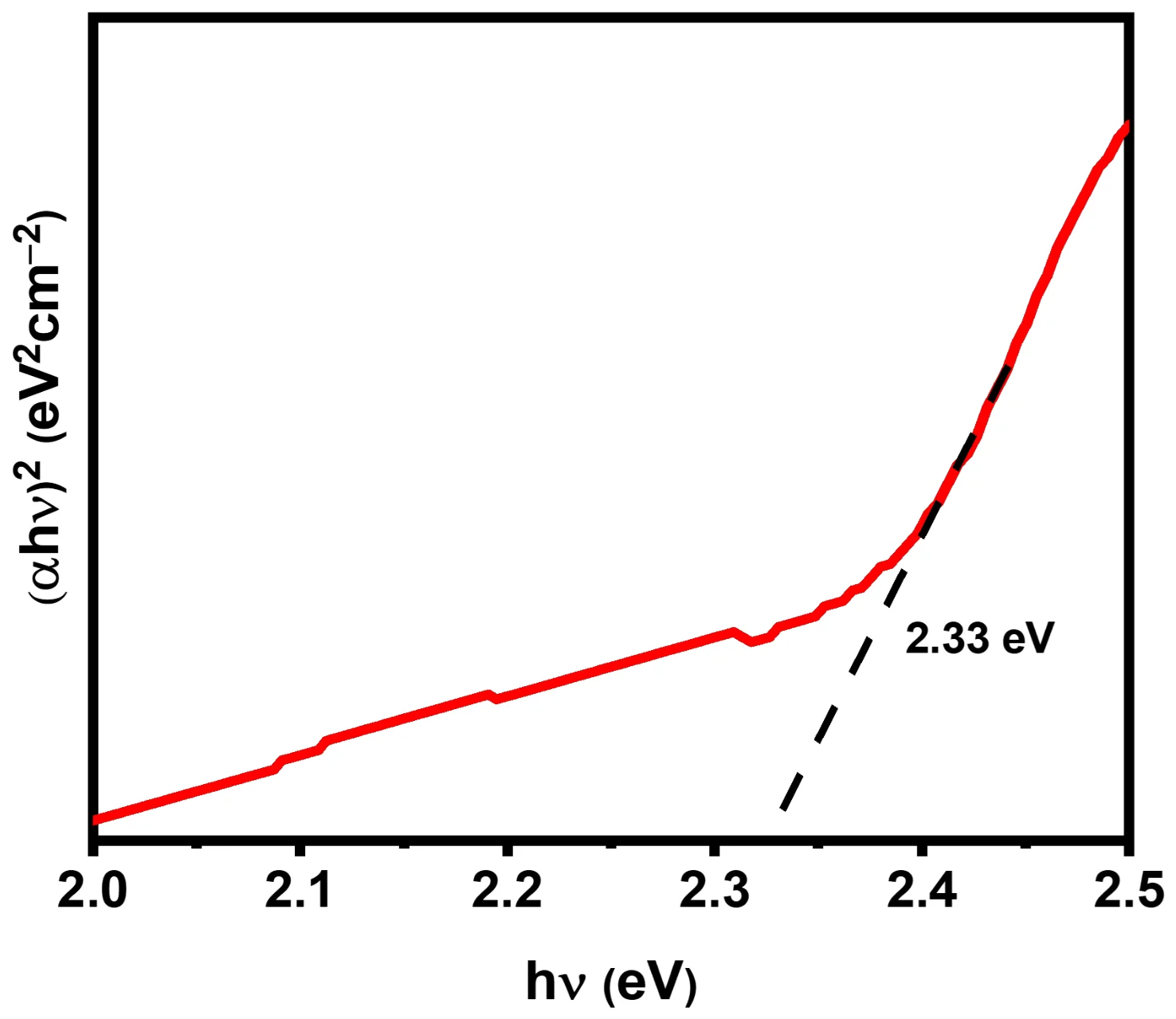
Optical band gap estimation of Zn/Fe@C from UV–Vis analysis.

The optical band gap of Zn/Fe@C was estimated from the solid-state UV–Vis absorption data according to the Tauc procedure described in Section 2.3. Assuming a direct allowed optical transition, the linear region of the (αhv)^2^ versus photon-energy plot was extrapolated, yielding an estimated optical band gap of approximately 2.33 eV, as shown in Figure 5. The obtained value indicates that Zn/Fe@C exhibits an optical response within the visible-light energy range, which is consistent with its photocatalytic application under simulated solar irradiation. Direct-transition Tauc analysis has also been applied to Zn–Fe ferrite-based photocatalytic materials [36]. However, considering the multiphase nature of Zn/Fe@C identified by XRD and the assumption of a direct allowed transition in the present analysis, the obtained value should be regarded as an apparent optical band-gap estimate of the composite material rather than the band gap of an individual crystalline phase.

### 3.2. Photocatalytic H_2_O_2_ Production

#### 3.2.1. Effect of Different Photocatalysts

**Figure 6:**
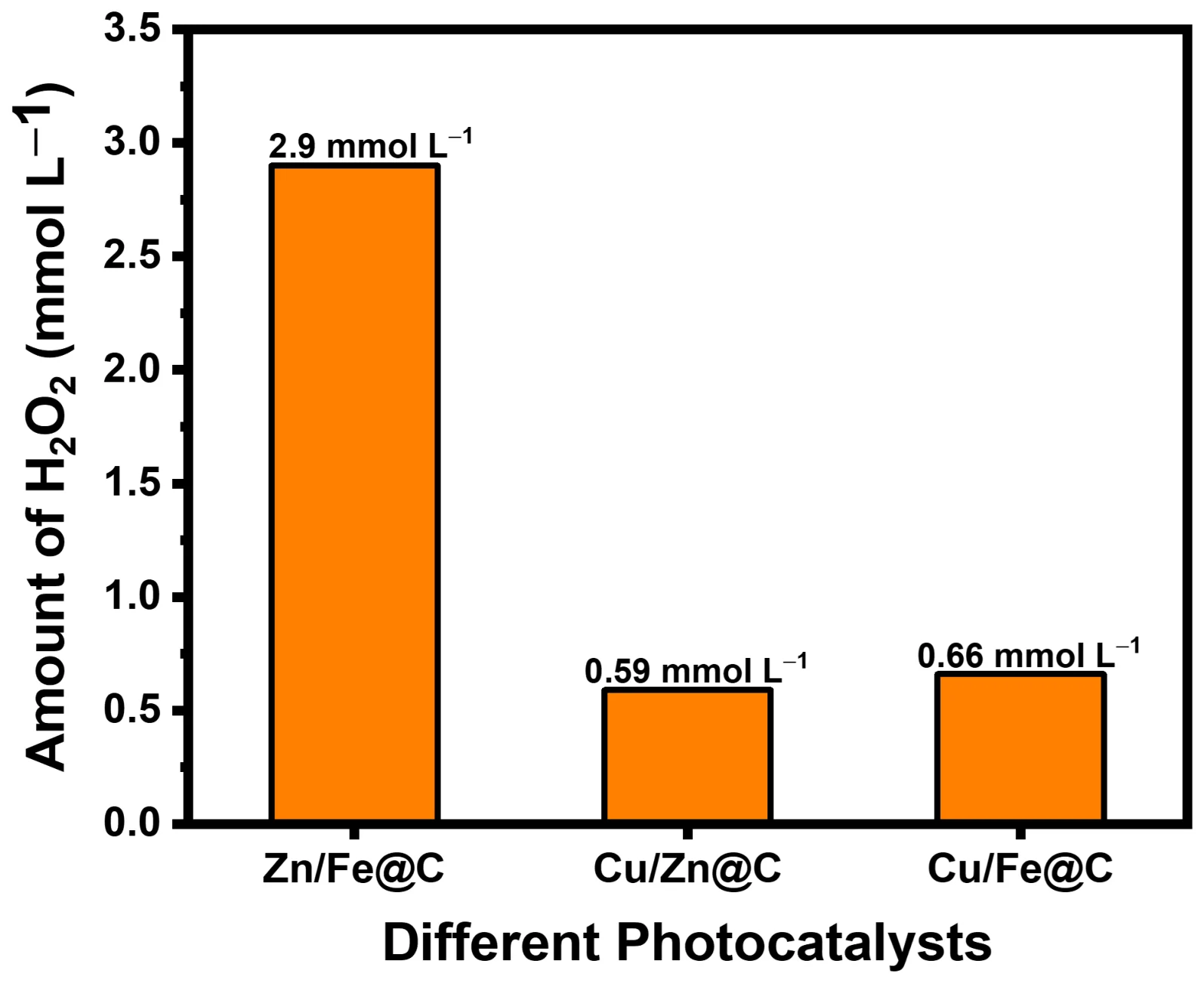
Effect of different photocatalysts on H_2_O_2_ production under simulated solar irradiation.

The photocatalytic performance of three photocatalysts, namely Zn/Fe@C, Cu/Fe@C, and Cu/Zn@C, was evaluated for H_2_O_2_ production under identical experimental conditions (Figure 6). All experiments were conducted using 2-propanol (2-PrOH) as the sacrificial agent, with a catalyst dosage of 20 mg and an irradiation time of 120 min. Among the tested materials, Zn/Fe@C exhibited the highest H_2_O_2_ production, reaching 2.9 mmol L^−1^ after 120 min, compared with 0.66 and 0.59 mmol L^−1^ for Cu/Fe@C and Cu/Zn@C, respectively. Accordingly, the H_2_O_2_ concentration obtained using Zn/Fe@C was approximately 4.4 and 4.9 times higher than those obtained using Cu/Fe@C and Cu/Zn@C, respectively. This substantial difference demonstrates that the nature and combination of the incorporated metal species strongly influence the photocatalytic H_2_O_2_ production performance. Photocatalytic H_2_O_2_ production is strongly influenced by the generation and separation of photogenerated charge carriers, interfacial electron transfer, and the adsorption and activation of molecular oxygen [37,38]. In particular, efficient utilization of photogenerated electrons can favor the oxygen reduction pathway toward H_2_O_2_ formation [28,29]. Therefore, the enhanced performance of Zn/Fe@C may be associated with more favorable charge-transfer and oxygen-reduction characteristics within the chitosan-derived carbon matrix. Carbon-based photocatalytic systems can facilitate the separation and transport of photoinduced charge carriers, thereby increasing their availability for surface redox reactions, including oxygen reduction [26]. However, since direct charge-carrier or surface-intermediate measurements were not performed in the present study, the superior activity of Zn/Fe@C is discussed as a possible consequence of these effects rather than being conclusively attributed to a specific Zn–Fe synergistic mechanism.

#### 3.2.2. Effect of Different Alcohols

**Figure 7:**
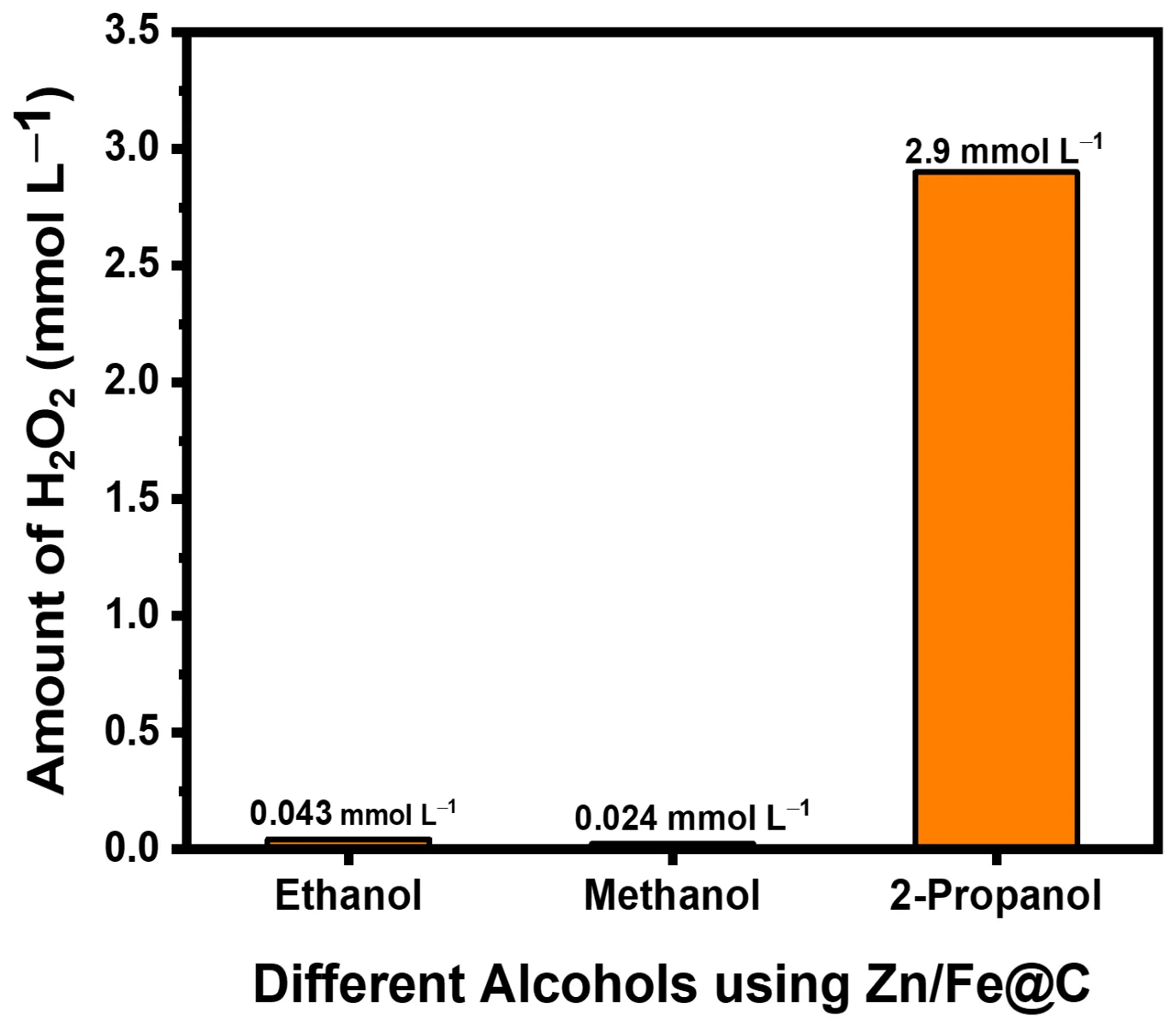
Effect of different alcohols on photocatalytic H_2_O_2_ production using Zn/Fe@C.

The influence of different alcohols on H_2_O_2_ production was investigated using Zn/Fe@C as the selected photocatalyst (Figure 7). Three alcohols, namely 2-PrOH, EtOH, and MeOH, were evaluated under identical conditions using a catalyst dosage of 20 mg. A pronounced dependence of H_2_O_2_ production on the type of alcohol was observed. Among the tested alcohols, 2-PrOH exhibited the highest H_2_O_2_ production, reaching 2.9 mmol L^−1^, whereas substantially lower concentrations of 0.043 and 0.024 mmol L^−1^ were obtained using EtOH and MeOH, respectively. Alcohols can act as sacrificial electron donors by consuming photogenerated holes, thereby suppressing electron–hole recombination and increasing the availability of photogenerated electrons for oxygen reduction toward H_2_O_2_ formation [7,39]. The strong dependence on alcohol type is consistent with previous photocatalytic studies demonstrating that the nature of the sacrificial electron donor can markedly influence H_2_O_2_ formation. In particular, the previously reported activity trend among aliphatic alcohols (2-PrOH > EtOH > MeOH) is consistent with the trend observed in the present study [39]. The superior performance of 2-PrOH may therefore be associated with differences in alcohol oxidation behavior, surface interactions, and hole-scavenging processes, which can influence interfacial charge transfer and the availability of electrons for O_2_ reduction [7,39]. Nevertheless, since alcohol oxidation intermediates and charge-transfer kinetics were not directly investigated, the enhanced H_2_O_2_ production observed with 2-PrOH cannot be conclusively attributed to a single mechanistic factor.

#### 3.2.3. Effect of Catalyst Loading

**Figure 8:**
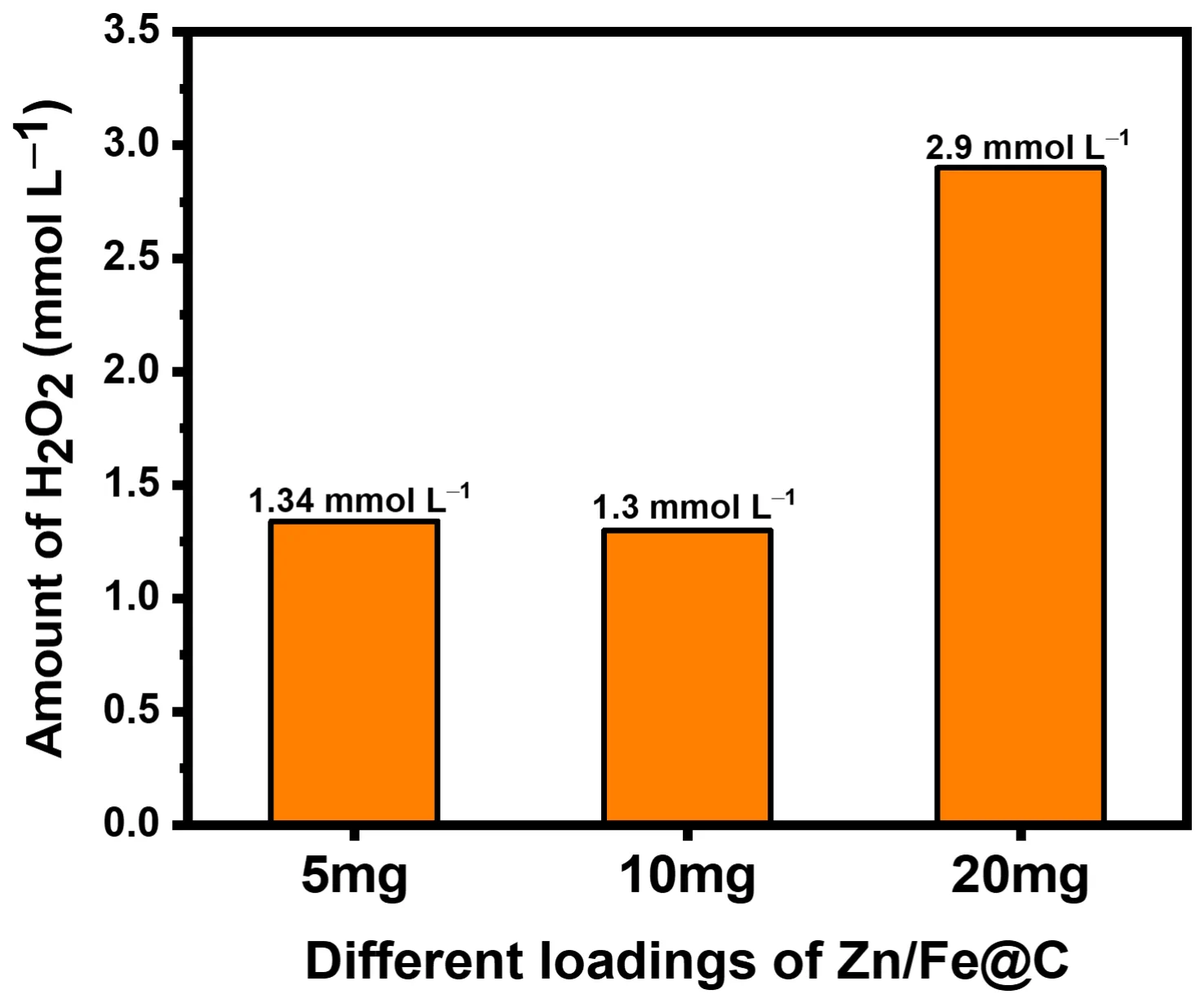
Effect of catalyst loading on H_2_O_2_ production over Zn/Fe@C.

The effect of catalyst loading on H_2_O_2_ production was studied using Zn/Fe@C in the presence of 2-PrOH (Figure 8). The catalyst dosage varied from 5 to 20 mg under identical reaction conditions. As shown in Figure 8, H_2_O_2_ concentrations of 1.34, 1.30, and 2.9 mmol L^−1^ were obtained using catalyst dosages of 5, 10, and 20 mg, respectively. The comparable H_2_O_2_ concentrations observed at 5 and 10 mg indicate that increasing the catalyst dosage within this range did not substantially affect H_2_O_2_ formation. In contrast, increasing the catalyst loading to 20 mg resulted in a pronounced enhancement in H_2_O_2_ production. The enhanced H_2_O_2_ production at 20 mg may be associated with the greater availability of photocatalytically active surface sites, providing more reaction sites for interfacial processes involved in H_2_O_2_ formation. Catalyst loading is an important parameter in photocatalytic H_2_O_2_ production and can substantially influence the amount of H_2_O_2_ generated [16]. Within the investigated range, 20 mg provided the highest H_2_O_2_ concentration and was therefore selected as the preferred catalyst loading for H_2_O_2_ production.

### 3.3. Photocatalytic Dye Degradation

#### 3.3.1. Effect of Different Dyes

**Figure 9:**
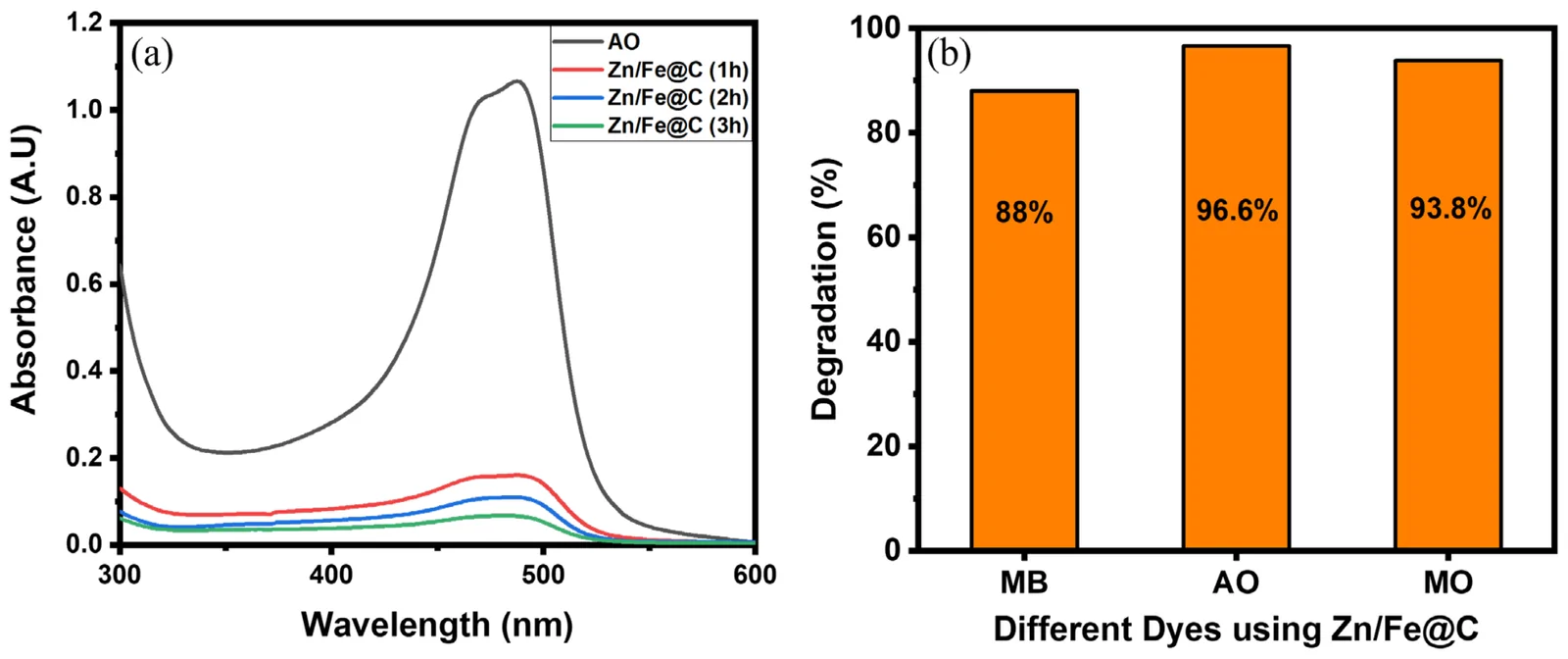
(**a**) UV-Vis spectrum of photocatalytic degradation of AO using Zn/Fe@C; (**b**) different dyes over Zn/Fe@C under visible light irradiation.

The photocatalytic activity of the Zn/Fe@C photocatalyst was evaluated toward the degradation of three representative dyes, namely MB, AO, and MO, under identical conditions (initial concentration 0.08 mmol L^−1^, catalyst dosage 5 mg, irradiation time 3 h) (Figure 9). Among the investigated dyes, AO exhibited the highest degradation efficiency (96.6%), followed by MO (93.8%) and MB (88%). The different degradation efficiencies observed under identical experimental conditions indicate that the photocatalytic response is dependent on the nature of the dye. Differences in molecular structure, ionic characteristics, and interactions between the dye molecules and the photocatalyst surface can influence adsorption behavior and subsequent photocatalytic degradation [40]. The high removal efficiency observed for AO suggests favorable interaction of AO with the Zn/Fe@C photocatalytic system. Previous studies have shown that AO can interact effectively with ZnO/carbon-containing surfaces through mechanisms such as electrostatic interactions, hydrogen bonding, and π–π interactions [41]. However, because dye-specific adsorption and surface-charge measurements were not performed in the present study, the differences among AO, MO, and MB cannot be conclusively attributed to a single adsorption or molecular factor.

#### 3.3.2. Effect of Catalyst Loading

**Figure 10:**
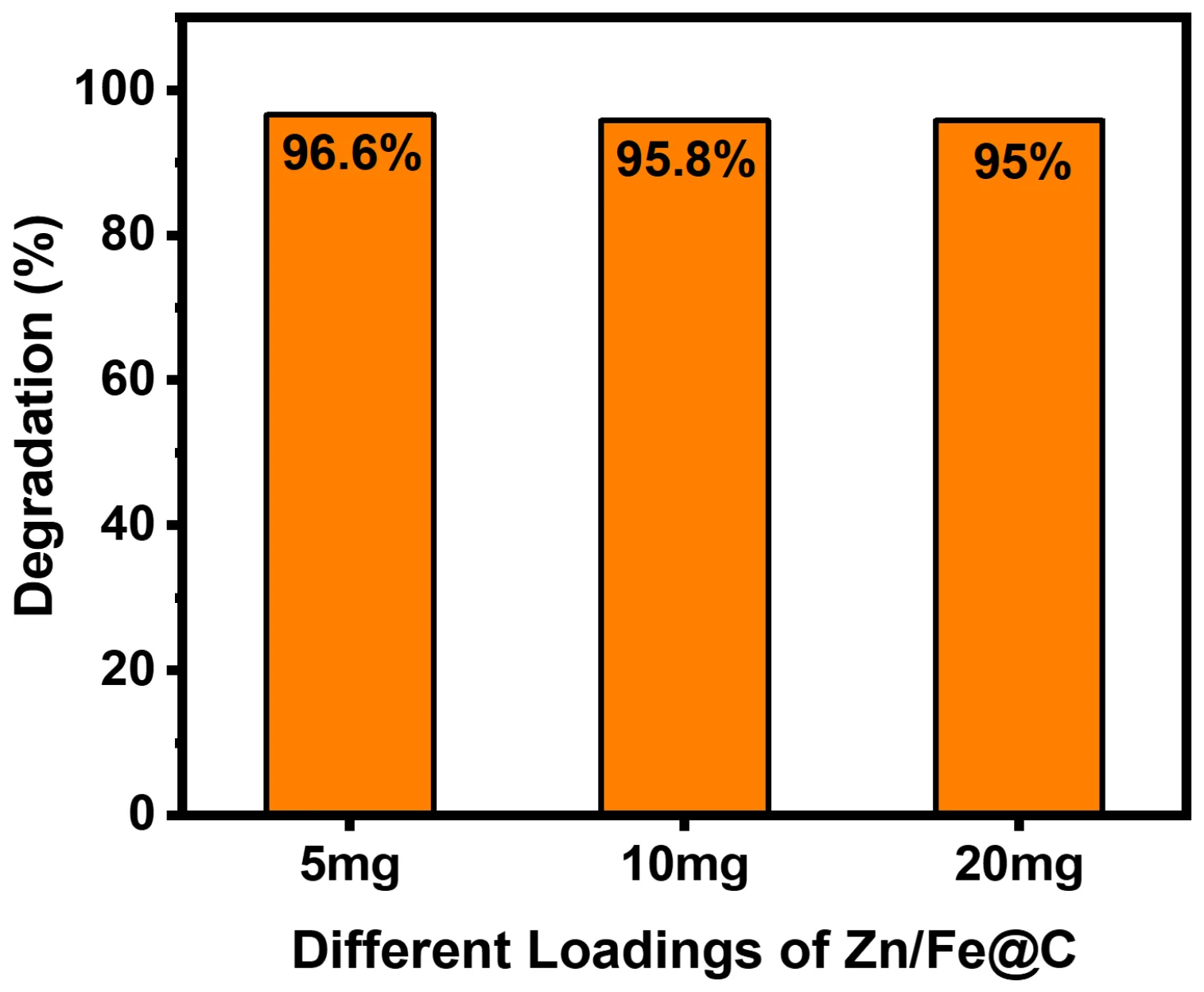
Effect of Zn/Fe@C catalyst loading on photocatalytic degradation efficiency of AO.

The influence of catalyst loading on the photocatalytic degradation of AO was investigated by varying the amount of Zn/Fe@C from 5 to 20 mg, while maintaining an initial AO concentration of 0.08 mmol L^−1^ under identical experimental conditions (Figure 10). The highest degradation efficiency was obtained using 5 mg of Zn/Fe@C (96.6%), followed by slightly lower efficiencies of 95.8% and 95.0% at catalyst loadings of 10 and 20 mg, respectively. Thus, increasing the catalyst loading beyond 5 mg did not provide any further improvement in AO degradation under the investigated conditions.

Although increasing the amount of photocatalyst generally provides a greater number of available active sites, excessive catalyst loading can adversely affect photocatalytic performance by increasing particle agglomeration and light scattering, thereby limiting effective light utilization [42]. Such effects may contribute to the slight decrease in degradation efficiency observed at higher Zn/Fe@C loadings. Nevertheless, given the relatively small differences in degradation efficiency among the three catalyst dosages, this interpretation should be considered a possible explanation rather than definitive evidence of particle aggregation or light-shielding effects. Within the investigated range, 5 mg was therefore selected as the preferred catalyst loading for subsequent AO degradation experiments.

#### 3.3.3. Dark Experiment

**Figure 11:**
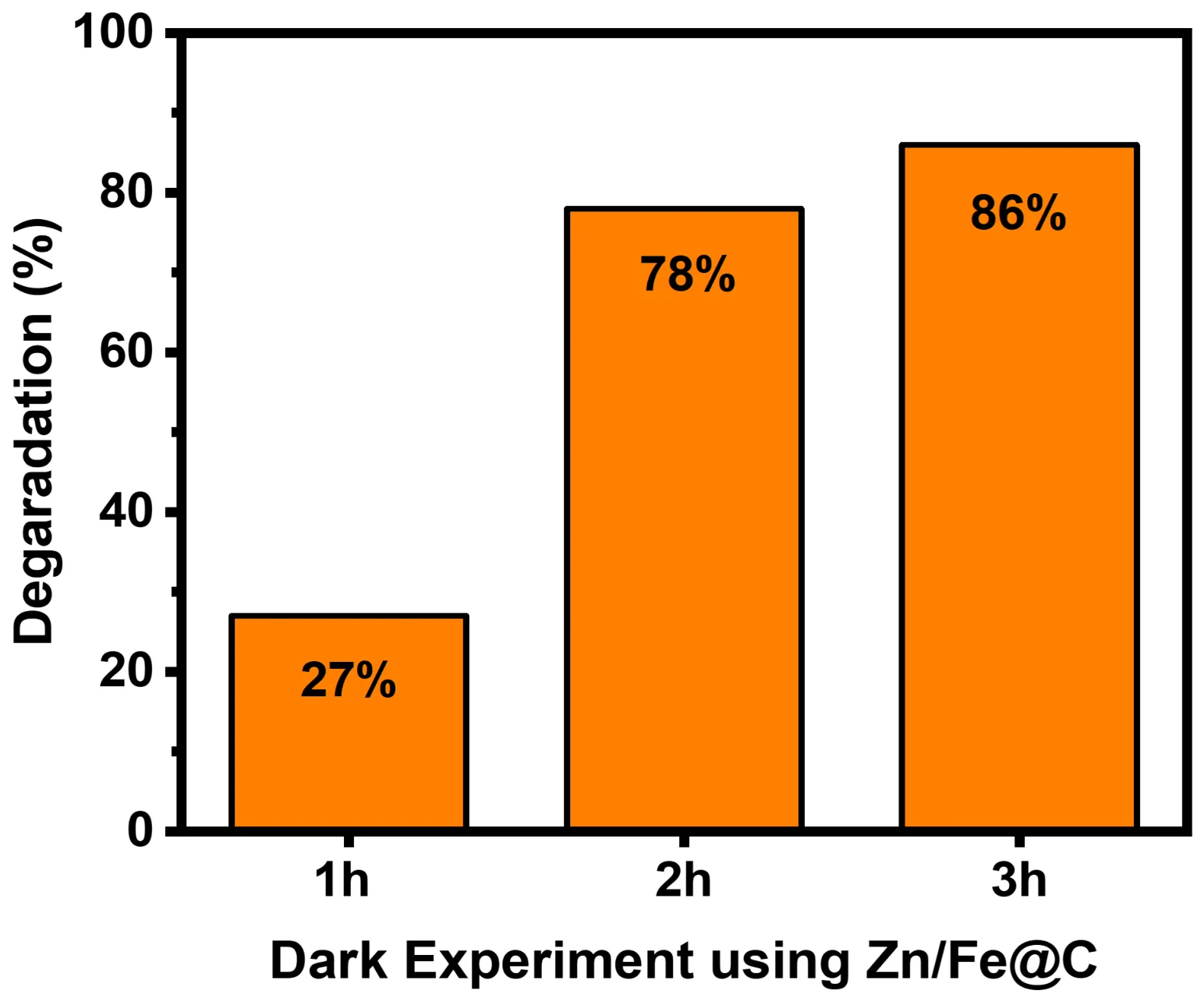
Time-dependent adsorption of AO using Zn/Fe@C under dark conditions.

To evaluate the contribution of adsorption to dye removal, a control experiment was conducted using AO in the presence of Zn/Fe@C under dark conditions (Figure 11). The adsorption experiment was performed for 3 h, corresponding to the duration of the photocatalytic degradation experiments. The AO removal efficiency increased from 27% after 1 h to 78% after 2 h and reached 86% after 3 h. These results demonstrate a substantial adsorption contribution of Zn/Fe@C toward AO. The strong adsorption behavior is consistent with previous studies reporting favorable interactions between AO molecules and ZnO/carbon-containing surfaces through mechanisms such as electrostatic interactions, hydrogen bonding, and π–π interactions [41].

The adsorption rate decreased toward the end of the investigated period, as indicated by the smaller increase in AO removal between 2 and 3 h compared with the earlier interval. This behavior suggests that adsorption was approaching a plateau; however, complete adsorption equilibrium cannot be conclusively confirmed within the investigated 3 h period. Under light irradiation, the AO removal efficiency reached 96.6% under comparable conditions, indicating an additional light-induced contribution beyond adsorption alone. Accordingly, AO removal by Zn/Fe@C is interpreted as a combined adsorption–photocatalytic process rather than being attributed exclusively to photocatalytic degradation.

#### 3.3.4. Effect of Metal Precursor Concentration

**Figure 12:**
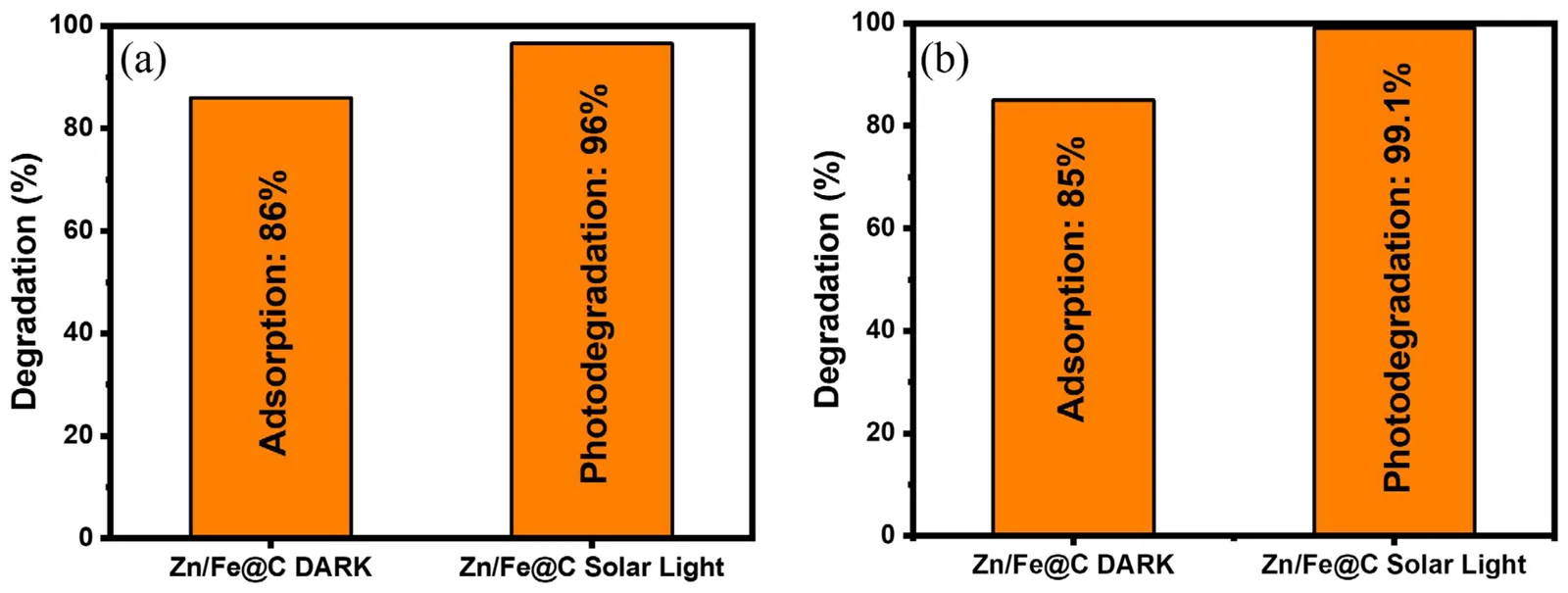
Effect of metal precursor concentration on AO removal performance over Zn/Fe@C: (**a**) adsorption and removal under light irradiation using the photocatalyst prepared at 3 mol L^−1^ and (**b**) adsorption and removal under light irradiation using the photocatalyst prepared at 5 mol L^−1^.

The effect of metal precursor concentration on the performance of Zn/Fe@C was investigated by increasing the precursor concentration from 3 mol L^−1^ to 5 mol L^−1^ (Figure 12a,b). Under dark conditions, comparable AO adsorption efficiencies were obtained for the photocatalysts prepared at 3 mol L^−1^ (86%) and 5 mol L^−1^ (85%), indicating that increasing the precursor concentration did not substantially enhance the adsorption contribution. In contrast, under light irradiation, the AO removal efficiency increased from 96.0% for the 3 mol L^−1^ sample to 99.1% for the 5 mol L^−1^ sample. The comparable adsorption efficiencies, together with the higher removal observed under irradiation for the 5 mol L^−1^ sample, suggest that the improvement cannot be explained by enhanced AO adsorption alone and may involve an increased light-induced photocatalytic contribution. Previous studies on Zn–Fe-based photocatalysts have demonstrated that variations in metal precursor composition during synthesis can significantly influence the resulting photocatalytic performance [43]. Increasing the metal precursor concentration in the present system may alter the amount, distribution, or chemical environment of Zn- and Fe-containing species within the carbon matrix, thereby influencing the photocatalytic behavior of the resulting material. However, because quantitative metal-loading and charge-carrier measurements were not performed for the two samples, the specific origin of the enhanced performance at 5 mol L^−1^ cannot be conclusively established. Therefore, the results demonstrate that precursor concentration influences the overall performance of Zn/Fe@C, with the 5 mol L^−1^ sample exhibiting the highest AO removal under irradiation.

#### 3.3.5. Reusability Assessment

**Figure 13:**
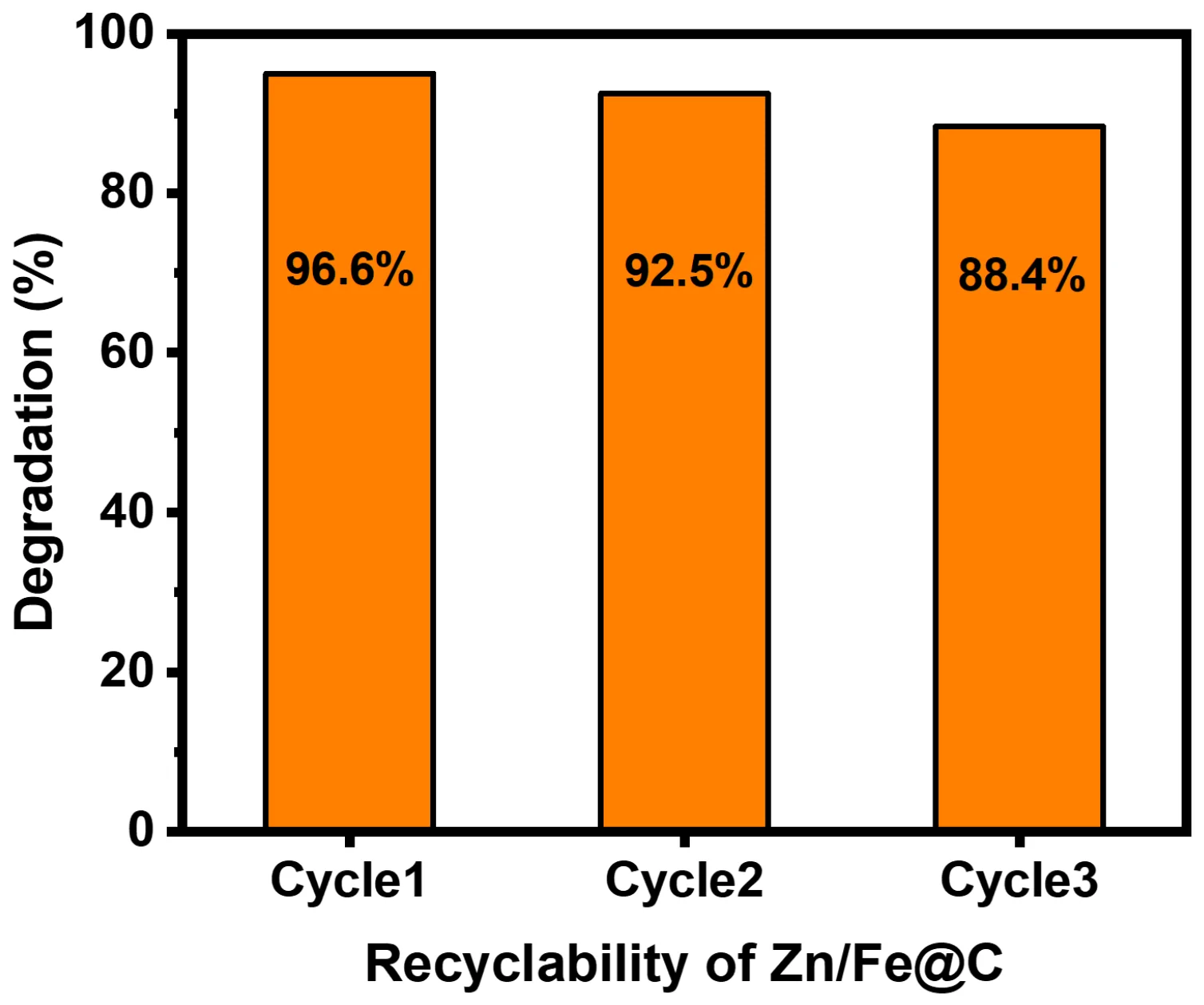
Reusability of Zn/Fe@C over three consecutive cycles.

The reusability of Zn/Fe@C was evaluated over three consecutive cycles using AO as the model dye (Figure 13). The AO removal efficiency gradually decreased from 96.6% in the first cycle to 92.5% in the second cycle and 88.4% in the third cycle. Nevertheless, Zn/Fe@C retained a relatively high removal efficiency after three consecutive cycles, demonstrating its potential for repeated use under the investigated conditions. The gradual decline in removal efficiency during successive cycles may arise from several factors commonly associated with photocatalyst reuse. These include partial blockage or deactivation of accessible active sites by adsorbed dye molecules or reaction intermediates, changes in the catalyst surface during repeated use, particle aggregation, and incomplete recovery or loss of catalyst during the separation and washing steps [44,45]. Possible leaching of active metal species may also contribute to activity loss during repeated use. Such effects could progressively reduce the number of accessible adsorption and photocatalytic sites available in subsequent cycles. However, these processes were not directly investigated in the present study and should therefore be considered possible explanations rather than confirmed causes of the observed decrease.

Although Zn/Fe@C retained an AO removal efficiency of 88.4% after the third cycle, the cycling experiment alone does not conclusively establish the structural or morphological stability of the photocatalyst. Post-use characterization, such as FTIR, XRD, or morphological analysis, would be required to determine whether structural or surface changes occur during repeated use. Therefore, the present results demonstrate promising short-term reusability of Zn/Fe@C, while further post-reaction characterization and extended cycling experiments would be valuable for establishing its long-term structural stability.

### 3.4. Possible Photocatalytic Mechanism

**Scheme 4:**
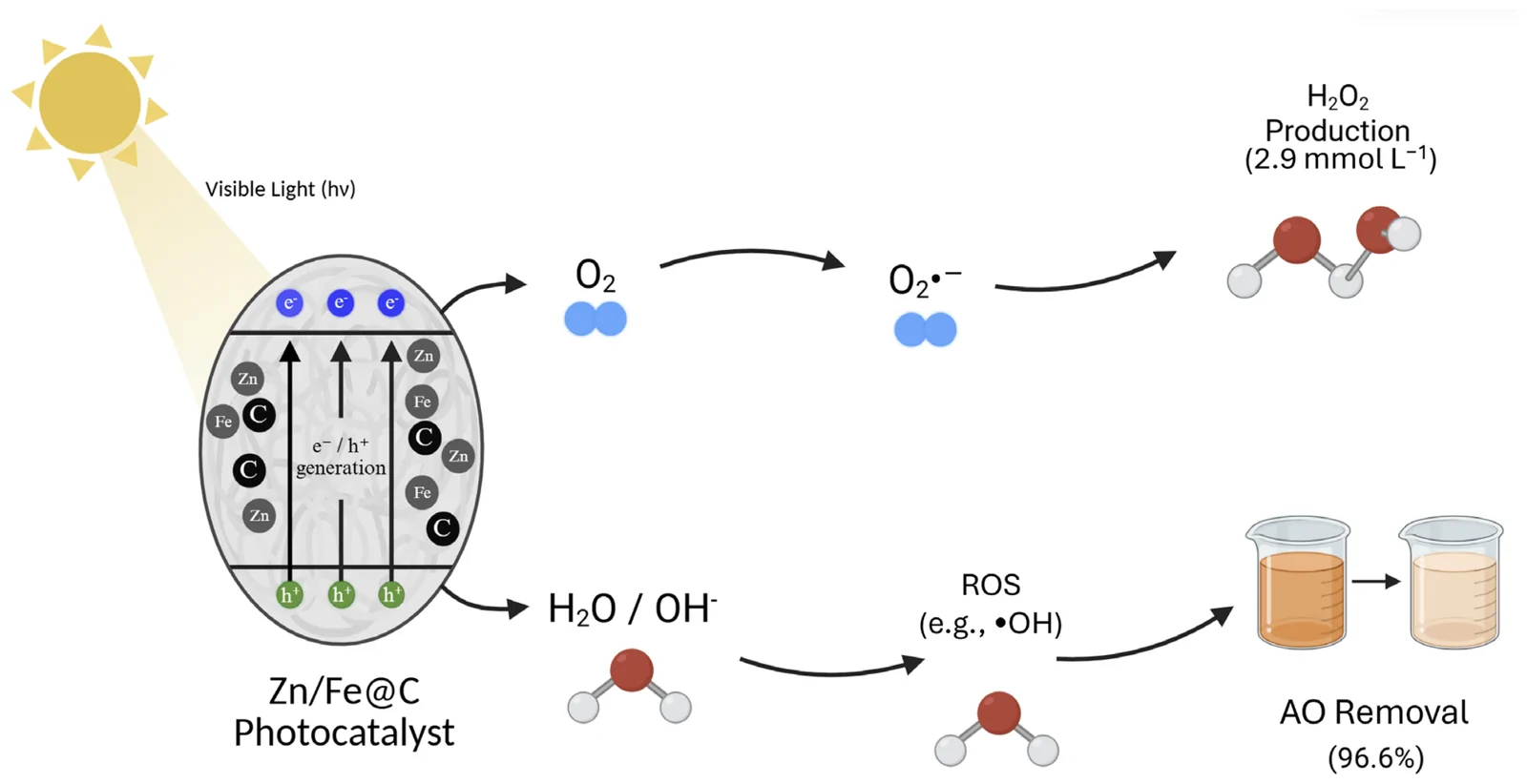
Proposed photocatalytic mechanism over Zn/Fe@C under visible light irradiation, illustrating electron–hole generation, oxygen reduction to H_2_O_2_, and dye degradation via reactive oxygen species.

The possible photocatalytic mechanism over the Zn/Fe@C photocatalyst under simulated solar irradiation is illustrated in Scheme 4. Upon irradiation, the photocatalyst can be photoexcited owing to its estimated band gap energy of 2.33 eV, resulting in the generation of photogenerated electrons (e^−^) and holes (h^+^):

**Zn/Fe@C + hν → e^−^ + h^+^**

The photogenerated electrons may participate in the reduction of dissolved molecular oxygen. H_2_O_2_ formation can proceed through a sequential oxygen-reduction pathway involving superoxide radicals (O_2_•^−^), as represented by the following equations, or through an overall two-electron oxygen reduction pathway [36]:

$$O_{2}+e^{-}\to O_{2}^{•-}$$

$$O_{2}^{•-}+{2H}^{+}+e^{-}\to H_{2}O_{2}$$

The overall reaction can be expressed as:

$$O_{2}^{•-}+{2H}^{+}+{2e}^{-}\to H_{2}O_{2}$$

The enhanced H_2_O_2_ production observed in the presence of 2-propanol (2-PrOH) may be related to its role as a sacrificial hole scavenger, which can consume photogenerated holes and thereby reduce electron–hole recombination, increasing the availability of electrons for oxygen reduction [46]. For dye removal, photogenerated holes and reactive oxygen species generated under irradiation may participate in oxidative reactions with the dye molecules. Hydroxyl radicals (•OH) may potentially be generated through reactions involving photogenerated holes and surface-bound H_2_O/OH^−^ species and subsequently contribute to dye oxidation. However, because radical-scavenging or spectroscopic measurements were not performed to directly identify the reactive species involved, the dominant oxidative species responsible for dye removal cannot be conclusively assigned in the present study. Similarly, the observed decrease in dye concentration should not be interpreted as complete mineralization because mineralization products were not directly quantified.

The enhanced H_2_O_2_ production observed in the presence of 2-propanol (2-PrOH) may be related to its role as a sacrificial hole scavenger, which can consume photogenerated holes and thereby reduce electron–hole recombination, increasing the availability of electrons for oxygen reduction [46]. For dye removal, photogenerated holes and reactive oxygen species generated under irradiation may participate in oxidative reactions with the dye molecules. Hydroxyl radicals (•OH) may potentially be generated through reactions involving photogenerated holes and surface-bound H_2_O/OH^−^ species and subsequently contribute to dye oxidation. However, because radical-scavenging or spectroscopic measurements were not performed to directly identify the reactive species involved, the dominant oxidative species responsible for dye removal cannot be conclusively assigned in the present study. Similarly, the observed decrease in dye concentration should not be interpreted as complete mineralization because mineralization products were not directly quantified.

Overall, the observed photocatalytic behavior of Zn/Fe@C may involve photogenerated charge carriers, oxygen-reduction pathways leading to H_2_O_2_ formation, and reactive oxygen species contributing to dye removal under simulated solar irradiation. Nevertheless, the proposed pathways in Scheme 4 should be regarded as plausible mechanistic routes based on experimental observations and established photocatalytic principles rather than directly confirmed elementary reaction steps.

## 4. Conclusions

In this study, a chitosan-derived Zn/Fe-based photocatalyst Zn/Fe@C was successfully synthesized via a green and facile route involving aqueous processing and subsequent thermal treatment. Structural characterization confirmed the formation of a predominantly crystalline, multi-phase oxide material, while morphological analysis revealed a rough and heterogeneous surface with aggregated features that are favorable for photocatalytic applications. Elemental analysis further verified the successful incorporation of Zn and Fe species within the chitosan-derived framework. The photocatalytic performance of the prepared material was evaluated through hydrogen peroxide (H_2_O_2_) production and dye degradation experiments. Among the tested photocatalysts, Zn/Fe@C exhibited superior activity, achieving the highest H_2_O_2_ production performance. The use of 2-propanol as a sacrificial agent significantly enhanced the process, while increasing catalyst loading improved H_2_O_2_ generation due to the availability of additional active sites. In dye degradation experiments, the photocatalyst demonstrated high efficiency toward the removal of different organic dyes, with acridine orange showing the highest degradation efficiency. The effect of catalyst loading revealed that lower dosages provided optimal performance, which was attributed to improved light penetration and reduced aggregation effects. Control experiments confirmed that photocatalysis was the dominant mechanism over adsorption. Furthermore, increasing the metal precursor concentration enhanced photocatalytic activity, indicating the beneficial role of higher active site density. The photocatalyst also exhibited good reusability over multiple cycles with only a slight decrease in performance. The estimated band gap of 2.5 eV supports the ability of the material to operate effectively under visible light irradiation. Overall, the results demonstrate that the developed Zn/Fe@C photocatalyst is a promising candidate for sustainable photocatalytic applications, offering efficient H_2_O_2_ production and effective degradation of organic pollutants under mild conditions.

## Data Availability

Data is contained within the article.
